# Rapid genomic convergent evolution in experimental populations of Trinidadian guppies (*Poecilia reticulata*)

**DOI:** 10.1002/evl3.272

**Published:** 2022-01-18

**Authors:** Mijke J. van der Zee, James R. Whiting, Josephine R. Paris, Ron D. Bassar, Joseph Travis, Detlef Weigel, David N. Reznick, Bonnie A. Fraser

**Affiliations:** ^1^ Biosciences University of Exeter Exeter EX4 4QD United Kingdom; ^2^ Department of Biology Williams College Williamstown Massachusetts 01267; ^3^ Department of Biological Science Florida State University Tallahassee Florida 32306; ^4^ Department of Molecular Biology Max Planck Institute for Developmental Biology Tübingen 72076 Germany; ^5^ Department of Biology University of California, Riverside Riverside California 92521

**Keywords:** Convergent evolution, experimental evolution, guppies, *Poecilia reticulata*, population genomics, rapid evolution

## Abstract

Although rapid phenotypic evolution has been documented often, the genomic basis of rapid adaptation to natural environments is largely unknown in multicellular organisms. Population genomic studies of experimental populations of Trinidadian guppies (*Poecilia reticulata*) provide a unique opportunity to study this phenomenon. Guppy populations that were transplanted from high‐predation (HP) to low‐predation (LP) environments have been shown to evolve toward the phenotypes of naturally colonized LP populations in as few as eight generations. These changes persist in common garden experiments, indicating that they have a genetic basis. Here, we report results of whole genome variation in four experimental populations colonizing LP sites along with the corresponding HP source population. We examined genome‐wide patterns of genetic variation to estimate past demography and used a combination of genome scans, forward simulations, and a novel analysis of allele frequency change vectors to uncover the signature of selection. We detected clear signals of population growth and bottlenecks at the genome‐wide level that matched the known history of population numbers. We found a region on chromosome 15 under strong selection in three of the four populations and with our multivariate approach revealing subtle parallel changes in allele frequency in all four populations across this region. Investigating patterns of genome‐wide selection in this uniquely replicated experiment offers remarkable insight into the mechanisms underlying rapid adaptation, providing a basis for comparison with other species and populations experiencing rapidly changing environments.

**Impact Summary**

The genomic basis of rapid adaptation to new environments is largely unknown. Here, we take advantage of a unique replicated experiment in the wild, where guppies from a high predation source were placed into four low predation, previously guppy‐free localities. Earlier reports document census size fluctuations and rapid phenotypic evolution in these populations. We used genome‐wide sequencing to understand past demography and selection. We detected clear signals of population growth and bottlenecks at the genome‐wide level that matched the known history of population numbers. We then identified candidate regions of selection across the genome, some of which were shared between populations. In particular, using a novel multivariate method, we identified parallel allele frequency change at a strong candidate locus for adaptation to the low predation environment. These results and methods will be of use to those studying the growing number of examples of rapid evolution.

Historically, evolution in natural populations was thought to happen on a long timescale, one that could not be observed in real time (Gillespie 1994). However, recently it has become clear that evolutionary change can occur rapidly, on an ecological timescale and many studies have now shown that phenotypic traits can evolve substantially within a few generations (Endler 1980; Reznick et al. 1997; Losos 2011; Grant and Grant 2020). Studying the effect of rapid changes at the genomic level can provide insight into genetic constraints and mechanisms of evolutionary adaptation (Pascoal et al. 2014), which in turn could help develop conservation efforts tailored to a specific species or population, and ultimately help predict a population's response to future changes in their environment.

Population genetics theory and practice, however, often relies upon a drift‐dominant model of evolution with few hard sweeps, which is unlikely to be the case in rapidly evolving populations (Messer et al. 2016). First, rapid genetic adaptation is likely to occur through soft sweeps on standing genetic variation (SGV) because adaptation is most rapid when the beneficial alleles are already present in the populations, whereas in hard sweeps it takes time for beneficial de novo mutations to appear (Hermisson and Pennings 2005; Barrett and Schluter 2008). In a soft sweep, multiple haplotypes can rise to high frequency and as a result, the effect of selection on levels of diversity and the allele frequency distribution are usually less severe, making them difficult to detect (Garud et al. 2015). Evolution on ecological timescales is also predicted to involve polygenic trait architecture, leading to very subtle allele frequency changes across many loci (Jain and Stephan 2017; Höllinger et al. 2019), which will add to the detection difficulty. Additionally, incomplete sweeps have been documented in experimental populations (e.g., Orozco‐terWengel et al. 2012).

Besides the challenge of detecting soft sweeps, many studies of rapid evolution have been made in single populations. If genomic methods could be applied to case studies of multiple populations experiencing rapid convergent evolution, their power would be increased substantially. The occurrence of convergent evolution is considered strong evidence for natural selection, as processes other than selection (such as genetic drift and random mutations) are unlikely to result in the same evolutionary changes in independent populations. Here, following Lee and Coop (2017), we use the term convergence to define repeated, independent allele frequency changes, which includes both classically defined convergent and parallel evolution as it includes evolution on standing genetic variation and de novo mutations. By looking for replicated allele frequency change in convergently evolving populations, we should be able to distinguish random genomic changes from those caused by recent selection.

Here, we capitalize on a unique set of rapidly evolving replicated experimental populations of guppies (*Poecilia reticulata*) to investigate the early stages of adaptation. We first examine patterns of genetic variation with whole genome sequence data to understand the impacts that neutral processes may have had on the populations. Then, we investigate signals of selection across the genome and instances of genomic convergence among the four replicate populations with a combination of selection scans and a multivariate analysis of allele frequency change vectors.

## Methods

### THE TRINIDADIAN GUPPY SYSTEM AND EXPERIMENTAL DESIGN

The guppies in the Northern Range Mountains of Trinidad are a well‐known model for studying phenotypic evolution in wild and experimental populations, offering a powerful platform to investigate convergent genomic adaptation. The mountains are drained by a set of parallel rivers punctuated by waterfalls preventing upstream colonization by larger fish species, including the guppy predators, resulting in replicated gradients of environments from high‐predation (HP) to low‐predation (LP) environments within rivers (Reznick and Travis 2019). Therefore, a number of phenotypic differences associated with predation are consistent across drainages. For example, LP males are more colorful (Endler 1980) and mature at a large size and later age, and LP females give birth to fewer and larger young than their HP counterparts (Reznick and Endler 1982; Reznick and Bryga 1996; Magurran 2005). Many of the repeatable phenotypic differences between HP and LP populations are also heritable in laboratory experiments (Reznick and Endler 1982; Reznick and Bryga 1996).

**Figure 1:**
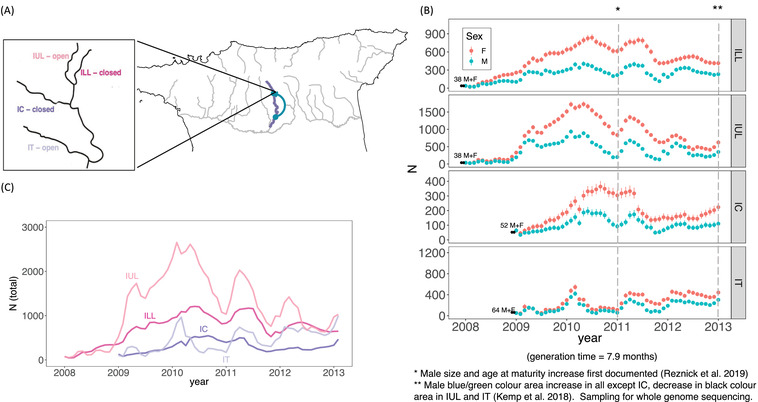
Summary of experimental populations. Map (A) highlights the Guanapo river with the sampled populations, and an inset shows the experimental rivers. “Open” indicates sites with manipulated canopies, “closed” indicates intact canopies. (B) Summary of mark‐recapture census data (from Reznick et al. 2019) per sex, annotated with introduction sizes (in black), and years where phenotypic evolution was documented. Estimates and confidence intervals are from a POPAN survival model implemented in program MARK. (C) Census data for all four populations plotted on the same axis.

Here, we report whole genome sequencing on four experimental populations that were initiated with founders from the same source population: Upper Lalaja (IUL), Lower Lalaja (ILL), Taylor (IT), and Caigual (IC). The common ancestors of the fish placed in these four locations were fish from a high predation site in the Guanapo River (GHP), which is downstream from the four populations in the same drainage. There were two independent collections of GHP fish, one per introduced pair, and the introductions were designed to introduce the same distribution of genetic variation into each member of a pair. ILL and IUL were introduced with much lower initial population densities due to fewer introduced fish and larger stream size than either IC and IT. IUL and IT had artificially thinned tree canopies (Fig. 1), which increased primary productivity (Kohler et al. 2012). Natural barriers restrict migration into the experimental populations.

A unique advantage of our experimental system is that both census population size and phenotypic change have been carefully monitored since their introduction, using monthly mark‐recapture data and annual common garden phenotypic evolution studies (second‐generation lab‐reared individuals; Reznick et al. 2019). All populations grew in their first year; ILL and ULL reached peak densities after 3 years and IT and IC reached peak densities after only 2 years (Fig. 1B, C; Reznick et al. 2019). All four populations also experienced fluctuations between dry and wet seasons. Population densities under thinned canopies (IUL and IT) fluctuated more than those under intact canopies (ILL and IC). These fluctuations were particularly strong in IT, where Reznick et al. (2019) observed the near extinction of the population during the rainy season in the first year and steep decline in the second year (Fig. 1C).

Phenotypic evolution in these populations has been well documented. Kemp et al. (2018) found that lab‐reared males had larger coverage of blue/green spots compared to the source population in all populations except for IC, and smaller black spots in IUL‐ and IT‐derived males (Fig. 1C). These changes reflect evolution after a release from heavy predation (Endler 1980). Reznick et al. (2019) showed that, by 2011, lab‐reared males from the four populations matured at a later age and larger size compared to those from the source population. This change reflects evolution in response to high population densities and high levels of intra‐ and interspecific competition (Bassar et al. 2013., 2017.; Potter et al. 2019). The evolutionary change in male age and size at maturity occurred at the same time, despite two different introduction times, likely because all four populations reached their peak densities in the same year (Reznick et al. 2019; Potter et al. 2021).

We sampled fish in 2013 for whole genome sequencing. Using a generation time of 7.9 months estimated from Potter et al. (2021) for ILL, this would be 6.1–7.6 generations since introduction. Recently, by surveying five pairs of naturally colonized HP‐LP populations, we found evidence for convergent evolution across the genome. Specifically, we identified a few strong candidates of adaptive loci shared among rivers and convergence at the genetic pathway level (Whiting et al. 2021a). This work can provide us with a list of candidate regions to compare to our experimental populations.

### SAMPLING, SEQUENCING, AND SNP CALLING

We sequenced approximately 20 individuals from each site (total *N* = 94, both males and females; Tables S1 and S2; Fig. 1C; mean coverage 9.0). Reads were mapped to the updated guppy genome (Fraser et al. 2020), SNPs were called using GATK (version 4.0.5.1), and were then phased following (Malinsky et al. 2018). For more details, see Supporting Information.

### GENOME‐WIDE DIVERSITY, POPULATION STRUCTURE, AND RUNS OF HOMOZYGOSITY

Principal Component Analysis (PCA) was performed on a linkage‐pruned VCF (–indep‐pairwise 50 5 0.2) in Plink version 1.90b6.7 (Chang et al. 2015) to assess population structure. Population‐specific summary statistics were calculated with PopGenome (nucleotide diversity [π], Tajima's *D*, and global *F*_ST_) (Pfeifer et al. 2014) and VCFtools version 0.1.16 (expected and observed heterozygosity, *H*_e_ and *H*_o_) (Danecek et al. 2011). Runs of homozygosity (ROH) were calculated for each individual with a sliding window approach with 50 SNPs per window using Plink (see Supporting Information for more details).

### SELECTION SCANS

Allele frequencies were calculated in VCFtools to investigate how many fixed differences exist between GHP and each introduced population, as well as allele frequency changes (ΔAF) between GHP and each of the introduced populations. This was done by calculating the change in minor allele frequency in nonoverlapping windows of 75,000 bp, chosen based on LD decay (Fig. S1). The introduced populations have only recently been established and we found only slight differences in divergence between these populations and the GHP source (see *Results*). Therefore, we chose to use two statistics based on haplotype homozygosity that are more suitable for detecting very recent directional selection. Our reasoning was that strong selection will sweep a haplotype to high frequency before recombination or mutation will break up the haplotype. First, we used cross‐population extended haplotype homozygosity (XP‐EHH), which was developed to detect selective sweeps that are nearly fixed in one population but still polymorphic in the entire metapopulation (Sabeti et al. 2007), akin to a haplotype‐based *F*_ST_ outlier scan. Second, we used iHH12, which similarly uses extended haplotype homozygosity but within a population and combines the top two most frequent haplotypes into a single haplotype, making it more effective at detecting soft sweeps (Garud et al. 2015; Torres et al. 2018). More details can be found in *Methods* in Supporting Information.

### MULTIVARIATE ANALYSIS OF PARALLEL ALLELE FREQUENCY CHANGES

We adapted De Lisle and Bolnick's (2020) multivariate approach to investigate parallel allele frequency changes in all four populations. Instead of using phenotypic change vectors, we applied the method to vectors of allele frequency change between the source population and each of the introduced populations. Briefly, we divided each chromosome into nonoverlapping windows of 200 SNPs, and calculated a matrix (*X*) of allele frequency changes (ΔAF) per SNP between the founder and each introduced population:

$$X=\begin{array}{ccc} \Delta AF_{1,1} & \cdots & \Delta AF_{n,1}\\ \vdots & \ddots & \vdots \\ \Delta AF_{1,m} & \cdots & \Delta AF_{n,m} \end{array},$$

where *n* is the number of SNPs in a window (in this case 200) and *m* is the number of introduced populations (in this case four). Each row was normalized to unit length (length = 1). For each of these matrices, we then computed the correlation matrix describing correlations of normalized allele frequencies among the vectors of each HP‐LP pair, by multiplying matrix *X* with its transpose:

$$C=XX^{T}$$

Eigen decomposition of each C matrix produces a distribution of eigenvalues. Distributions with excessive variance explained by the first eigenvector suggest a shared direction of allele frequency changes through multivariate space among all population pairs. This direction of change could be parallel (along the same axis in the same direction) or antiparallel (along the same axis in different directions). We then created a null‐distribution to determine which windows show extreme eigenvalues. We randomly sampled windows of an equal number of SNPs (*N* = 200) along the genome, and allowed for a random start position for windows. For each null permutation, all individual IDs were shuffled and allele frequency vectors recalculated between random pairs. This null permutation approach therefore captures potential bias associated with features of the genomic landscape and the sampling design, but for randomized vector trajectories. We applied the same transformations described above to each window and ran 10,000 permutations. Windows were identified as outliers if they were above 99.9% of our randomized null. Finally, we investigated where the allele frequency changes are parallel and where they are antiparallel by examining the loading of each population on the first eigenvector. Lineages with the same sign loading can be interpreted as evolving in the same direction, and lineages with opposite signs are evolving antiparallel. The code to perform this method is available as an R package: AF‐vapeR (https://github.com/JimWhiting91/afvaper). The method is fully described and validated with simulated data in Whiting et al. (2021b). We focused only on the first eigenvector here to highlight cases where all four populations were evolving in parallel.

### SIMULATION METHODS

We used forward‐in‐time simulations using a Wright‐Fisher model with SLiM3 (Haller and Messer 2019) to examine expected neutral allele frequency trajectories in each introduced population based on census sizes since founding. The goal here was to quantify the expected effect of drift on allele frequency change within each introduced population. From this, we can get a sense of how much of the observed differentiation between experimental populations and the founder might be expected under neutrality. We used Ne estimates from the source GHP population estimated previously (Whiting et al. 2021a) to initiate each experimental population. We then used census data to forward simulate each population 200 times. More information can be found in *Methods* in Supporting Information.

For each mutation, for each simulation, we calculated the selection coefficient based on a diploid single locus model, to standardize for starting allele frequency according to:

$$s=\frac{2}{\tau }In\left(\frac{p_{\tau }q_{0}}{p_{0}q_{\tau }}\right),$$

where τ is the number of generations between founding and sampling, and *p* and *q* are minor and major allele frequencies at founding (*p*_0_) and sampling (*p*_τ_). The aim here was to produce a neutral distribution of standardized allele frequency change under drift for each population to compare with observed standardized allele frequency change, calculated on the GHP minor allele, to determine whether these are significantly nonzero. For observed data, we assume the allele frequency observed in GHP is representative of the founding frequency given the large size of this population and short time between sourcing and sampling. Alleles are polarized to the GHP minor allele, so our standardized allele frequency change measures include information on direction and magnitude of change. We therefore summed these estimates within SNPs across populations, such that the summed value is maximized by allele frequency change that is of large magnitude and in the same direction.

## Results

### POPULATION STRUCTURE AND GENOME‐WIDE DIVERSITY REVEAL LIMITED DIVERGENCE BETWEEN THE EXPERIMENTAL POPULATIONS AND THEIR SOURCE

**Figure 2:**
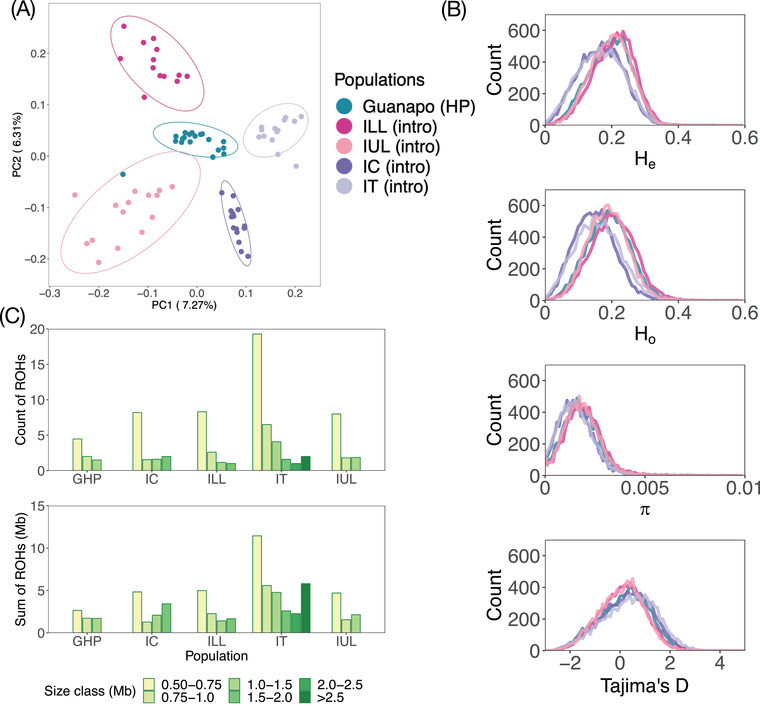
Population structure, runs of homozygosity, and neutral population statistics across the introduction sites and the HP source. PCA (A) with populations colored according to river; 75‐kb window‐based estimates of (B) expected heterozygosity (*H*_e_), observed heterozygosity (*H*_o_), nucleotide diversity (π), and Tajima's *D* (D) for each of the introduced populations and their HP source. (C) Number and sum of runs of homozygosity (ROH) in different size classes, per population.

Experimental populations showed limited divergence from their source (GHP; Fig. 2A). PC1 and PC2 accounted for 7.3% and 6.3% of the variation, respectively (Fig. 2A); PC1 separates the two pairs of populations introduced on different years, whereas PC2 separates populations in different directions. Clustering by introduction year is expected because each pair of populations was founded by individuals collected from the founding site on different years. PC3 and PC4 did not further separate populations and all other individual PCs accounted for <5% of the variation (Fig. S2). When compared to natural populations across the Northern Range described in Whiting et al. (2021a), the experimental populations show very limited divergence from their source (GHP) (Fig. S3).

Pairwise *F*_ST_ was low between introduced populations and their source (median *F*_ST_ = 0.013–0.023), with the strongest difference being that between IT and GHP (Table S3). This higher *F*_ST_ between IT and GHP, despite the observation of relatively low divergence between the two on PC2, can be explained by the low within‐population variation of IT, resulting in GHP variants that are more likely to have been lost in IT. This is consistent with the expected loss of genetic diversity caused by the population crash that occurred in IT during the first wet season. Among the experimental populations, IC, IUL, and ILL were more genetically distinct from IT than they were from one another (pairwise FST; Table S3). Guppies from populations initiated in the same year were more similar to each other than guppies from populations initiated in different years (Table S3). This is again a consequence of there having been two independent collections of GHP fish, one per introduced pair, and the way introductions were designed to introduce the same distribution of genetic variation into each member of a pair.

The introduction of guppies from GHP to ILL, IUL, IC, and IT led to very minor changes in genetic diversity within the introduction populations (Fig. 2B; Table S4). IT and IC experienced a decrease in *H*_e_ compared to GHP (15.8% and 15.5%, respectively, *P* < 0.0001 for both, Mann‐Whitney *U* test), whereas IUL experienced very slight increase of *H*_e_ (0.5%, *P* = 0.07) and a significant increase in ILL (5.4%, *P* < 0.0001). Similarly, IT and IC experienced a decrease in *H*_o_ (17.4% and 22.6%, respectively, *P* < 0.0001). ILL also decreased in *H*_o_ (6.8%, *P* < 0.00) but IUL increased (1.1%, *P* = 0.005). Median nucleotide diversity (π) decreased in IT and IC (15.1% and 14.4%, respectively, *P* < 0.0001, for both) and was slightly, but significantly, increased in ILL and IUL (8.0% and 2.6%, *P* < 0.0001 and *P* = 0.002, respectively). Tajima's *D* was shifted to a more positive value in IC and IT compared to GHP (Fig. 2B; Table S4; *P* < 0.0001). This suggests a loss of rare alleles compared to the source, which is consistent with a sudden population contraction. In ILL and IUL, Tajima's *D* has decreased slightly compared to GHP, but stayed positive (Fig. 2B; Table S4; *P* < 0.0001), implying an increase of rare alleles that could be related to rapid population expansion.

### RUNS OF HOMOZYGOSITY PROVIDE EVIDENCE OF RECENT INBREEDING AND A BOTTLENECK IN IT

The patterns of ROH are consistent with known history: GHP, the source population, had the lowest number of ROH (*N* = 83) and IT, which experienced a population crash in the first year after initiation, had nearly three times more ROH than the other introduced populations (*N* = 423; Table S5). In general, we found ROH of >0.5 Mb in 93 of the 94 individuals, with one individual in GHP lacking the ROH fitting requirements we set a priori. The other three experimental populations had comparable numbers of ROH (IUL [*N* = 151], IC [*N* = 150], and ILL [*N* = 142]).

We also observed variation among populations in the maximum length of ROH. IT was the only population with ROH >2 Mb (Fig. 2C; Table S5), consistent with the known bottleneck after initiation in this population. ILL, IUL, and IC have more ROH and a larger proportion of their genome covered in ROH than GHP, suggesting there may have been a limited bottleneck effect of the initial collection (Fig. 2C).

Inbreeding coefficients (*F*_ROH_) were significantly higher in IT compared to the other introduced populations (Table S5; *P* < 0.0001, Mann‐Whitney *U* test), again confirming recent inbreeding in this population. The remaining three introduction populations did not significantly differ from each other (ILL‐IC: *P* = 0.274, ILL‐IUL: *P* = 0.650, and IUL‐IC: *P* = 0.624, Mann‐Whitney *U* test), but did have slightly higher values compared to GHP (GHP‐ILL: *P* < 0.0001, GHP‐IUL: *P* = 0.002, and GHP‐IC: *P* = 0.002, Mann‐Whitney *U* test).

### THERE ARE STRONG SIGNALS OF SELECTION IN ALL FOUR POPULATIONS DESPITE LIMITED DIVERGENCE GENOME‐WIDE

Mean allele frequency change was low in all populations (Table S6). There were no fixed differences in allele frequency between GHP and any of the four introduction populations. Of the SNPs that were the minor allele in GHP, fewer became fixed in ILL and IUL than in IC and IT (*N* = 97 in ILL, *N* = 102 in IUL, *N* = 385 in IC, and *N* = 469 IT; Table S6). None of these fixed SNPs were shared among all four populations.

**Figure 3:**
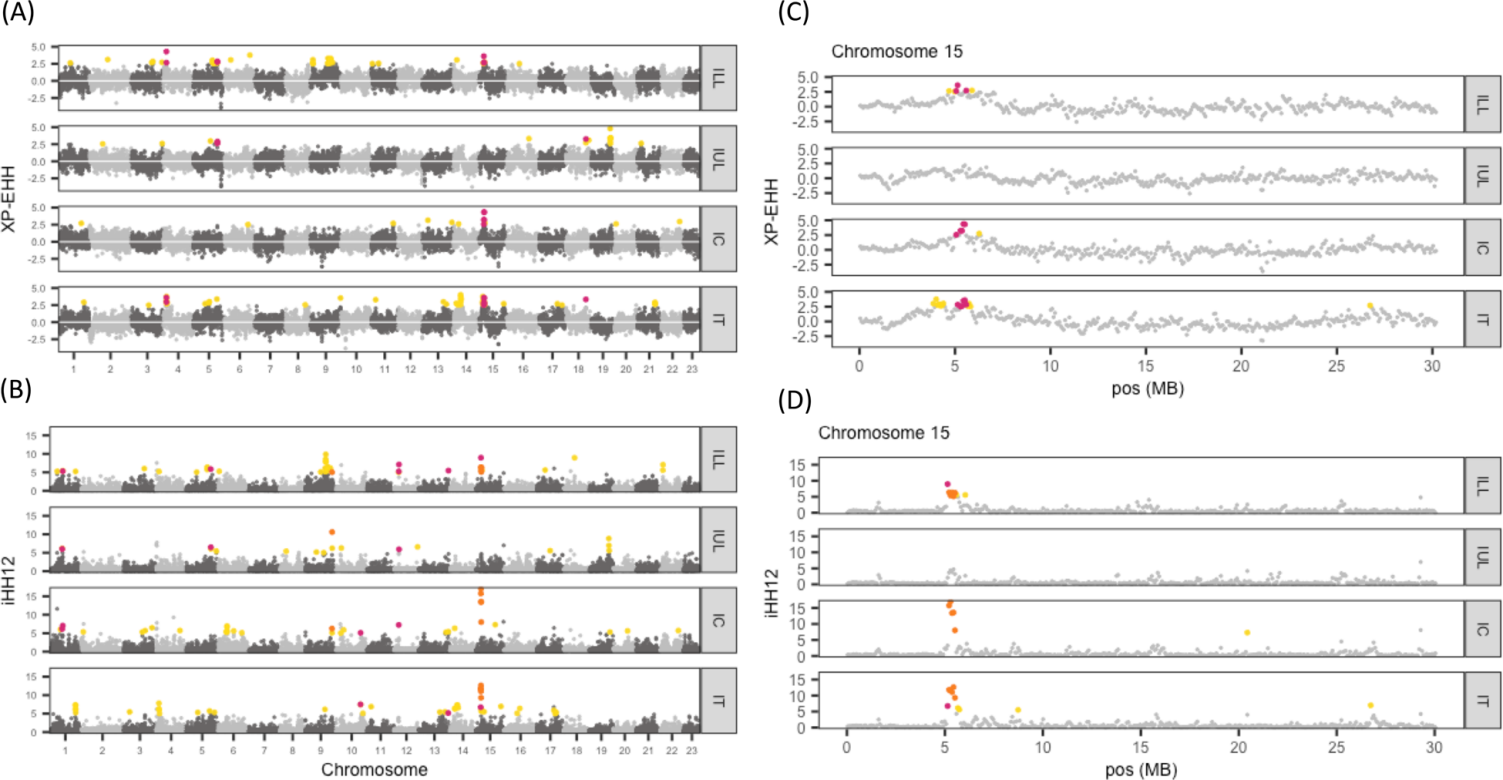
Haplotype genome scans (A) XP‐EHH and (B) iHH12 for each introduced population across the genome. Chromosome 15 (C) XP‐EHH and (D) iHH12. Yellow points indicate unique outlier windows among the populations, pink points show overlapping outlier windows in two of the four populations, and orange points indicate windows overlapping in three of the four comparisons; there was no overlap among all four comparisons. Outliers are defined as windows with XP‐EHH values >2.5 and iHH12 values >5.

We analyzed 9804 windows of 75 kb length using XP‐EHH and iHH12 to search for regions under selection in the experimental populations. For the XP‐EHH statistic, IT had the most outlier windows (*N* = 50), followed by ILL (*N* = 39), IUL (*N* = 17), and finally IC (*N* = 14) (Fig. 3A; Tables S7–S10). For iHH12, IT again had the most outliers (*N* = 41), followed by ILL (*N* = 39), IC (*N* = 36), and IUL (*N* = 19) (Fig. 3B; Tables S11–S14).

We found a ∼5‐Mb region on chromosome 15 that was a strong candidate for selection in three of the four introduced populations. This region overlapped in both statistics in three populations and had the highest iHH12 scores in two of the populations (Fig. 3C, D). For XP‐EHH, only pairwise overlaps were observed but the overlapping outliers for IC, IT, and ILL pairs all involved a small region on chromosome 15 (5,100,000–5,625,000 bp) (Fig. 3C; Table S15). Among the pairwise overlaps, IC‐IT and ILL‐IT overlapped more often (*N* = 4, for both), followed by ILL‐IUL (*N* = 2), with no overlap between IUL and IC (Table S15).

Similarly, for iHH12, there was little overlap in outliers, and no outlier windows were common to all four comparisons. However, among the three‐way overlapping outliers, ILL‐IC‐IT overlapped the most (*N* = 5, expected *N* ≤ 1; Table S16) and all overlapping windows were located consecutively on chromosome 15 (Fig. 3D; 5,175,001–5,550,000 bp). ILL‐IUL‐IC had two overlapping windows, one on chromosome 9 (28,500,001–28,575,000 bp) and one on an unplaced scaffold 000083F_0.2 (150,001–225,000 bp). The remaining three‐way comparisons had no overlapping outlier windows. Among the pairwise overlapping outliers, comparisons including IUL showed fewer overlapping outlier windows than the other comparisons. ILL‐IC and ILL‐IT had the most overlapping windows (*N* = 9 for both), followed by IC‐IT (*N* = 6), IUL‐IC (*N* = 5), ILL‐IUL (*N* = 4), and no overlap between IUL and IT. Taken together, shared signals of selection are not driven by introduction year or canopy thinning.

### ALLELE FREQUENCIES CHANGED IN PARALLEL FASHION IN THE INTRODUCED POPULATIONS IN A FEW CANDIDATE LOCI

**Figure 4:**
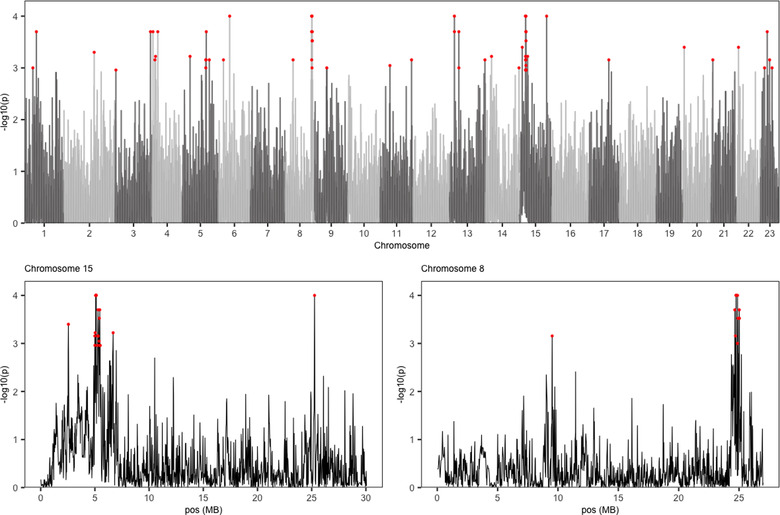
Eigenvector analysis. Transformed *P*‐values for eigenvector 1 across the genome. (B) Chromosome 15. (C) Chromosome 8. Red points indicate those at 99.9% quantile of the null distribution.

Our multivariate approach revealed that the four populations exhibited parallel changes in allele frequency in both previously identified candidate loci and newly identified loci. We identified 65 outlier windows spread over 17 of the 23 chromosomes on the first eigenvector, using the 99.9% quantile cutoff from the randomized allele frequency matrices (Fig. 4A; Table S17). Seventeen of these windows occurred in the candidate region of convergent evolution on chromosome 15 (4,998,794–5,492,324 bp; Fig. 4B). Another cluster of three outlier windows was on chromosome 8 (24,647,480–25,034,422 bp; Fig. 4C), which had not been identified previously (gene annotation in Table S18). Starting allele frequency and allele frequency change were similar between the chromosome 8 and chromosome 15 region, meaning allele frequency does not account for their differences in detection (Fig. S4). All populations had eigenvector loadings of the same sign and similar size for all of these outlier windows (Table S17). All four populations thus experienced parallel allele frequency changes along the same axis and in the same direction. Information on the second eigenvector can be found in Supporting Information (Table S19).

**Figure 5:**
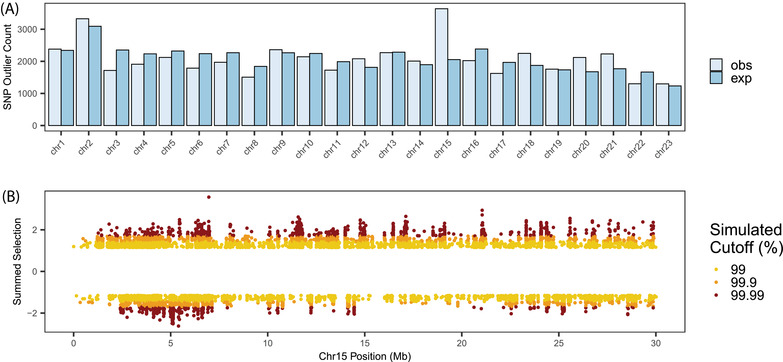
Forward simulated standardized allele frequency change, as estimated by selection coefficients based on neutral evolution and census size compared to observed selection coefficients. (A) Across the genome. (B) Across chromosome 15. Bars across the genome represent the expected number of outlier SNPs above the simulated 99.9% cutoff given the relative size of each chromosome compared with the observed number. Points along chromosome 15 show the per‐SNP summed selection coefficients, representing the summed estimates of standardized allele frequency change between the source (GHP) and each introduced population. Values are maximized when standardized allele frequency change is large in all populations and allele frequency change is in the same direction (same sign for estimated selection coefficient). Colors indicate SNPs with summed selection coefficients above the 99% (yellow), 99.9% (orange), and 99.99% quantiles defined by the neutral simulations based on census size estimates.

Simulated allele frequency change distributions (standardized as selection coefficients) were similar to observed distributions in each of the experimental populations (particularly ILL and IUL), which means that much of the genome‐wide allele frequency change from GHP to each experimental population is broadly in‐line with neutral expectations (*Results* in Supporting Information and Fig. S5). Drift was apparent in all simulated populations under neutrality, and was strongest in IC (mean absolute selection coefficient under neutrality = 0.128), followed by IT (mean = 0.114), ILL (mean = 0.111), and IUL (0.107). No simulated SNP became fixed in an introduced population after starting as the minor allele in the founding population. We then sought to identify SNPs where the summed strength of co‐directional allele frequency change (summed within SNPs across all four introduced populations) exceeded the simulated, neutral expectation. This analysis highlighted 47,577 SNPs across all chromosomes that exceeded the simulated 99.9% expectation. Chromosome 15 exhibited a particularly striking excess of outlier SNPs relative to the expectations given its size (Fig. 5A), in agreement with previous selection scans. This excess on chromosome 15 implies allele frequency change here is likely nonneutral, in comparison to the rest of the genome. Of particular note were a collection of outlier SNPs in the region at 5 Mb that suggest selection against the GHP minor allele in all four introduced populations (Fig. 5B). The strongest SNP signal here (chr15:5403609), and therefore the upper limit of selection for this region, exhibited selection coefficients of IC = −0.67, IT = −0.72, ILL = −0.62, and IUL = −0.62 acting on the GHP minor allele.

### THERE WERE CONVERGENT SIGNALS OF SELECTION IN ALL FOUR POPULATIONS IN A CANDIDATE REGION ON CHROMOSOME 15

Closer examination of allele frequency change between the GHP source and introduction populations confirmed parallel evolution at our candidate loci. When comparing allele frequency change among all four populations and their source, SNPs within our candidate chromosome 15 region showed similar change. They were more strongly correlated to each other than an equal‐sized random sample of SNPs (*P* < 0.0001 for all comparisons, co‐correlation analysis [Diedenhofen and Musch 2015]). This is also true for pairwise comparisons with IUL, for which we found no outlier regions on chromosome 15 (Fig. S6), confirming that all four populations are evolving along the same axis in this region.

Taking all our selection analyses together, we can identify candidate genes involved in rapid adaptation to the LP environment (Fig. S7; Table S20). Overlapping the outliers of eigenvector 1 and XP‐EHH on chromosome 15 highlighted a roughly 4‐Mb region from 5,066,474 to 5,456,741 bp (Fig. S7) that encompasses 11 candidate genes (Table S20). However, the summed selection simulation analysis highlights a much smaller region. Therefore, this region shows the strongest evidence for parallel allele frequency change and falls within the cadherin‐1 and B‐cadherin coding regions. Genes in the cadherin family have been implicated in regulating pigment cell migration and are involved in cell‐cell adhesion interactions (Fukuzawa and Obika 1995; Nishimura et al. 1999). The cadherin signaling pathway is also enriched for signatures of selection in all paired comparisons of naturally colonized HP and LP populations (Whiting et al. 2021a). Further studies, such as gene knockout experiments, could help identify the exact role of the cadherin genes in this process.

## Discussion

Our four rapidly evolving, experimental populations of guppies revealed limited genome‐wide differentiation from their source population. Despite these small genome‐wide changes, we uncovered strong local signals of selection in all four populations. Using a combination of haplotype genome scans, forward simulations, and a newly developed multivariate approach, we found evidence for parallel change among the four descendants at candidate loci. Our multivariate approach revealed more subtle parallel changes in allele frequency compared to the genome scans. Our results will be of interest to those looking to detect recent, ecological timescale changes in demography and selection at the genomic scale.

We identified a strong candidate for rapid adaptation to the new LP environment on chromosome 15, where we found signatures of selection in three of our four populations and parallel change in all four populations. This region has been previously identified as a candidate for convergent evolution in a RADSeq study in both natural and long‐term experimental populations (specifically in the HP‐LP Oropuche, Arima river pairs, and an introduction LP population in the Aripo River) (Fraser et al. 2015). Further, this same region was found to be a candidate for selection in the HP‐LP Tacarigua and Oropuche River population pairs using whole genome sequencing (Whiting et al. 2021a). By examining the haplotypes and allele frequency change among natural HP‐LP pairs with our results here, we can compare the trajectory of change between the natural and experimental populations. The experimental populations are evolving toward the haplotype present in the natural LP population within its drainage (Guanapo; GLP), but there is not a shared common LP haplotype across all LP populations (Fig. S8). Further, applying eigenanalysis (AF‐vapeR) to the natural population dataset reveals that this region is an outlier on eigenvector 2, indicating multiple axes of HP to LP AF change across northern Trinidad (Whiting et al. 2021b). Biologically, this is consistent with different but functionally similar variants. This pattern in turn is consistent with the strong iHH12 signal found in the experimental populations, which combines the two most frequent haplotypes to detect soft sweeps. On the basis of this evidence, we hypothesize that a candidate “LP” gene is harbored within this region.

Rapid convergent genomic adaptation, specifically at quantitative traits, is predicted to often occur through small shifts in allele frequency (Barrett and Schluter 2008). With our new multivariate approach, we were able to detect small but parallel shifts in 65 windows. Many of the phenotypic traits under selection in LP in guppies are likely to have polygenic control. If this is the case, then simultaneous selection on standing genetic variation at many loci would cause subtle shifts of allele frequencies at individual loci (Pritchard et al. 2010). These factors would result in small parallel shifts in allele frequency rather than complete sweeps at individual loci. A similar polygenic response was found in an artificial evolution experiment for size in Atlantic silversides (*Menidia menidia*), where many unlinked loci showed parallel change in addition to change in a large haplotype present in the founder (Therkildsen et al. 2019). A polygenic response in experimental evolve resequence populations may be widespread (Barghi et al. 2020).

Many of the parallel evolving windows lacked a strong haplotype signal in the genome scan measures and none of these windows were outliers in all four populations, which can be explained in several ways. First, it is possible the variance on this axis was constrained for reasons other than positive selection. For example, background selection (BGS) could cause correlated differentiation landscapes among the populations, which results in a constrained axis of allele frequency changes (Burri 2017). However, others (Matthey‐Doret and Whitlock 2019; Stankowski et al. 2019) have shown with simulations that when divergence times are short, BGS is unlikely to generate the correlated differentiation landscapes we report here. Other processes, such as positive selection, are the more likely cause of these patterns. Second, the strength of selection may have been relatively weak, causing time of fixation for a beneficial allele to be considerably longer. These weakly selected alleles would not have had time to generate extreme frequency differences in the introduced populations (Coop et al. 2009). However, because we detected parallel allele frequency change in all four populations, its actual variance relative to the rest of the genome is less important. Finally, haplotype‐based statistics rely on long maintained stretches of signal, whereas our multivariate method identifies regions of parallel shifts in allele frequency, for example, the outlier region on chromosome 15 was much larger than our region on chromosome 8 (Fig. S4) and therefore window size selection likely contributed to the differences in method detection. However, a balance must be struck between detection and the increased false positives associated with increased number of smaller sized windows, especially where recombination rates vary. We find no correlation with the multivariate approach with recombination (Table S21). Nor is the chromosome 15 candidate in a region of low recombination (Whiting et al. 2021c) or within population specific local PCA outliers (Fig. S9).

It is widely appreciated that demographic changes associated with changes in population size can create patterns that resemble the consequences of selection. Therefore, studies of rapid evolution ought also to explore past demographics. Unfortunately, most demographic inference methods are underpowered at recent timescales (e.g., PSMC, fastsimcoal2 [Li and Durbin 2011; Excofffier et al. 2021]) or small sample sizes (e.g., hap‐ibd [Zhou et al. 2020]), making such methods unreachable for rapid evolution studies on nonmodel organisms. Here, we had the advantage of comparing demographic inference to known census size fluctuations and can therefore offer insights on whether there is a single statistic most sensitive to recent demographic changes. Specifically, one of our populations, IT, went through a population crash post‐introduction (Reznick et al. 2019). Our results would thus be of interest to those interested in studying recent, perhaps human‐induced, population crashes.

Haplotype statistics were more sensitive to a recent population crash in IT than site frequency spectrum (SFS) methods. Although IT had a decrease in *H*_e_ and π compared to the source population, overall changes in allele frequencies were subtle. These differences were similar to IC, which experienced no large post introduction crash yet was introduced at a similarly high population density. However, IT was a clear outlier in length and number of ROHs, indicators of a recent bottleneck (Tajima 1989; Smith and Haigh 2007). All four experimental populations showed higher numbers of ROH than their source population, likely reflecting the founding bottleneck. Similarly, the effects of recent human bottlenecks were found in haplotype rather than SFS statistics (Gattepaille et al. 2013). Subsequent population growth was much less apparent on the genomic level. ILL and IUL, the two replicates initiated with lower population densities, ultimately grew to larger population sizes. They both experienced a slight increase in expected heterozygosity and nucleotide diversity and a reduced Tajima's *D* compared to the source GHP, which is indicative of populations that experienced rapid population expansion (Zenger et al. 2003; Ortego et al. 2007). However, again these signals were subtle or nonsignificant.

It could be expected that, as a result of the bottleneck, IT might have sustained a chance loss of genetic variation that caused it to have an altered evolutionary trajectory relative to the other three replicates. The natural population of guppies found downstream from the IT population had a higher mortality rate than IC in the first year. This elevated mortality has been attributed to a stronger influence of flood‐induced mortality during the wet season and perhaps disease (Dowdall et al. 2012; Fitzpatrick et al. 2014). Therefore, the selection regime in this particular river may be different than the others. However, we find no evidence for any associated difference in selection regime in IT. In our overlapping outlier analysis, comparisons including IT consistently had high numbers of overlapping outlier windows, suggesting IT was not following a different trajectory from the other three populations. Similarly, our AF‐vapeR analysis showed that IT was evolving in parallel with the other populations in our candidate windows with the other populations, evidenced by similar loadings (Table S17).

In conclusion, our analysis and results will be of use to the study of rapid evolution at the genomic level. We have the unique advantage of known, replicated episodes of population and adaptive phenotypic change that can ground our results. First, we are able to detect the effects of known population crashes and founding events across the genome. Next, we were able to detect strong candidates of selection and more subtle signatures of parallel change using a novel multivariate method. This method has the potential to detect convergent evolution in rapidly evolving populations, likely to involve more complex evolutionary responses, such as small parallel changes at many loci or changes along multiple vectors.

### CONFLICT OF INTEREST

The authors declare no conflict of interest.

### AUTHOR CONTRIBUTIONS

BAF, DNR, RB, JT, and DW conceived the idea of the study. DNR, RB, and JT conducted the initial introduction experiment. Genomic work and analysis were completed by MJvdZ, JRW, JRP, and BAF. Writing was done by all authors.

### DATA ARCHIVING

The data that support these findings are openly available at: European Nucleotide Archive (https://www.ebi.ac.uk/ena/browser/home)—reference numbers: PRJEB42705 (all introduction populations) and PRJEB10680 (GHP). All scripts and associated data are available on Github repository: mapping and SNP calling (https://github.com/josieparis/gatk‐snp‐calling); population genomics and haplotype scans (https://github.com/bfraser‐commits/Rapid_genomic_adaptation_guppies); the software for multivariate AF analyses (AF‐vapeR) (https://github.com/JimWhiting91/afvaper); and simulation analyses (https://github.com/JimWhiting91/fibr_simulations).

## Supporting information

supplementary Tables and Figures information

supplementary Tables and Figures information
