# Supplementary material for: Rapid genomic convergent evolution in experimental populations of Trinidadian guppies (Poecilia reticulata)
Source: Evol Lett. 2022 Jan 18;6(2):149–61. doi: 10.1002/evl3.272 (PMC8966473; doi:10.1002/evl3.272)
Supplement: Supplementary file 1 — supplementary Tables and Figures information [file EVL3-6-149-s001.docx]

Supplementary Files for:

# Rapid genomic convergent evolution in experimental populations of Trinidadian guppies (*Poecilia reticulata*)

This supplement contains:

Supplementary Methods

Supplementary Results

Figure S1: Linkage decay for each experimental population and their source.

Figure S2: PC3 and PC4 with populations coloured according to river.

Figure S3: Principal component analysis with natural populations described in [(Whiting et al., 2021a)](https://paperpile.com/c/U27TGp/pSKrq)

Figure S4: Allele frequency changes compared to eigenvalue p-values for outlier regions.

Figure S5: Summary of simulation analysis

Figure S6: Allele frequency changes in the outlier windows on chromosome 15

Figure S7: Comparing selection statistics in candidate region on chromosome 15

Figure S8: Genotype plots comparing experimental populations to naturally colonised populations in our candidate region on chromosome 15

Figure S9: LocalPCA (Li and Ralph 2019) analysis over chr15

Table S1: Summary of population information and sequencing data.

Table S2: Coverage per individual

Table S3: Pairwise global FST values among all six populations.

Table S4: Summary of population genetic statistics

Table S5: Summary of ROH

Table S6: Summary of allele frequency changes in the introduced populations

Table S7: xpEHH outlier windows for Lower Lalaja (ILL)

Table S8: xpEHH outlier windows for Upper Lalaja (IUL)

Table S9: xpEHH outlier windows for Caigual (IC)

Table S10: xpEHH outlier windows for Taylor (IT)

Table S11: iHH12 outlier windows for Lower Lalaja (ILL)

Table S12: iHH12 outlier windows for Upper Lalaja (IUL)

Table S13: iHH12 outliers for Caigual (IC)

Table S14: iHH12 outlier windows for Taylor (IT)

Table S15: Observed and expected overlapping windows for XP-EHH outliers

Table S16: Observed and expected overlapping windows for iHH12 outliers

Table S17: Outlier windows on eigenvector 1

Table S18: Genes in the identified outlier region on chromosome 8

Table S19: Outlier windows on eigenvector 2

Table S20: Genes in the identified outlier region on chromosome 15

Table S21: Enrichment of outlier windows with areas of high or low recombination

SUPPLEMENTARY METHODS:

*Genome sequencing and SNP calling*

Fish were stored in 95% ethanol at -20C prior to DNA extraction. Genomic DNA was extracted from caudal peduncle tissue using the Qiagen DNeasy Blood and tissue kit (QIAGEN; Hilden, Germany). Whole genome sequencing libraries were prepared following the Illumina TruSeq DNA sample preparation guide with approximately 250bp insert size. Eighty-six samples were sequenced by multiplexing 6-10 individuals per lane on an Illumina HiSeq 2000 and 3000.The remaining eight samples were sequenced using the Illumina HiSeq 4000 with a 150bp paired-end read metric.

Quality of paired-end reads was assessed with FastQC [(Andrews, 2010)](https://paperpile.com/c/U27TGp/yfWf) and adapters and low-quality bases removed with TrimGalore! [(Krueger, 2012)](https://paperpile.com/c/U27TGp/7rakR). Reads were aligned to the long-read, updated guppy reference genome [(Fraser et al., 2020)](https://paperpile.com/c/U27TGp/EDJqQ) using BWA-mem (v0.7.17) [(Li and Durbin, 2009)](https://paperpile.com/c/U27TGp/o38nq). Read groups were added and duplicate reads were removed before merging to produce final bams using picard v 2.06. Read quality was recalibrated using variants generated from high-coverage, PCR-free sequencing data [(Fraser et al., 2020)](https://paperpile.com/c/U27TGp/EDJqQ). GVCFs were obtained using GATK’s (v4.0.5.1) HaplotypeCaller and combined with GenomicsDBImport before genotyping with GenotypeGVCFs. Resulting variants were filtered on the basis of QD<2.0, FS>60.0, MQ<40.0, HaplotypeScore>13.0 and MappingQualityRankSum < -12.5 according to GATK best practices, and only bi-allelic sites were retained. Genotypes were removed if they had a depth <5x and sites were removed if missing in 50% of individuals. Finally, the population files were merged again and filtered for a minor allele frequency of >0.01. The final VCF file contained 6,510,265 SNPs.

For the haplotype analyses, we followed the double-phasing protocol as described in [(Malinsky et al., 2018)](https://paperpile.com/c/U27TGp/tKOBw): population VCF files were first phased per chromosome using Beagle (v5.0) [(Browning and Browning, 2007)](https://paperpile.com/c/U27TGp/a7rbH), followed by a second round of phasing with Shapeit2 (v2.r904) [(Delaneau et al., 2011)](https://paperpile.com/c/U27TGp/osnB9). Shapeit2 has an increased accuracy compared to beagle [(Delaneau et al., 2011)](https://paperpile.com/c/U27TGp/osnB9), but does not accept missing data. Therefore, we use beagle, which does accept missing data but has a high switch rate error [(Delaneau et al., 2011)](https://paperpile.com/c/U27TGp/osnB9), to create pre-phased VCF files that can be used as input for shapeit2.

*Population statistics*

Population specific summary statistics were calculated with PopGenome (nucleotide diversity (𝝅), Tajima’s D and global F_ST_) [(Pfeifer et al. 2014)](https://paperpile.com/c/dyAw9r/sEe7) and VCFtools v0.1.16 (expected and observed heterozygosity, He and Ho)[(Danecek et al. 2011)](https://paperpile.com/c/dyAw9r/ahwg). For each measure we took the mean per fixed non-overlapping 75Kb window. Nucleotide diversity was estimated for fixed size (i.e. 75kb) not per SNP. Genome-wide F_ST_ was calculated by taking the mean and median of window non-weighted estimates. Allele frequency was calculated in vcftools and the minor allele defined as the minor allele in the source population (GHP), unless otherwise stated.

*Runs of homozygosity*

Runs of homozygosity (ROH) were calculated for each individual with a sliding window approach with 50 SNPs per window using Plink. To minimize the detection of ROH that could occur by chance, the minimum number of SNPs needed to constitute a ROH (l) was estimated using the method proposed by [(Lencz et al., 2007)](https://paperpile.com/c/U27TGp/nL3ug). Additionally, each run had to be at least 500 kb long to exclude short, common ROH present in all individuals and populations. Finally, at most 1 heterozygous site per window was allowed. Runs of homozygosity were estimated for each individual separately, and resulting ROH were binned into four categories: 0.5-0.75 Mb, 0.75-1.0 Mb, 1.0-1.5 Mb, and >1.5 Mb. To calculate the genomic inbreeding coefficient F_ROH_, we used:

$$F_{ROH}=\frac{\Sigma L_{ROH}}{L_{auto}}$$

Where L_ROH_ is the total length of all of an individual’s ROH above a specified length threshold and L_auto_ is the length of the autosomal genome.

*Genome scans*

Both XP-EHH and iHH12 were calculated using Selscan (v1.2.0a) [(Szpiech and Hernandez, 2014)](https://paperpile.com/c/U27TGp/dM2U) using the default settings, except for a MAF filter of 0.01. The results were then normalised over 75kb windows using the script provided by Selscan and the mean was taken. XP-EHH outliers were identified by a value of XP-EHH > 2.5, and iHH12 outliers were identified as those windows with an absolute value of iHH12 > 5 in the introduced populations and an absolute value of <5 in the GHP source. iHH12 was computed per introduced population, not the two populations (ancestral + introduced).

The number of overlapping outliers among populations per measure was calculated using the R package SuperExactTest [(Wang et al., 2015)](https://paperpile.com/c/U27TGp/OqfKX). This package also calculates the expected number of overlapping windows for each set based on a hypergeometric distribution.

To assess the extent of parallel allele frequency changes in the chromosome 15 outlier region, we extracted allele frequency changes per SNP (ΔAF) among the SNPs in the XP-EHH and iHH12 outlier windows and made pairwise plots of ΔAF. We compared these values to values of ΔAF to a pool of equal size drawn randomly across the genome. Finally, we obtained gene annotations for the outlier region by aligning the region to the previously published female genome [(Künstner et al., 2016)](https://paperpile.com/c/U27TGp/5MhYP) and extracting guppy genes using Ensembl’s BioMart (release 102) [(Howe et al., 2021)](https://paperpile.com/c/U27TGp/rrJPX).

*Simulation methods and results*

We used forward-in-time simulations using SLiM3 [(Haller and Messer, 2019)](https://paperpile.com/c/U27TGp/tZkz) to examine expected neutral allele frequency trajectories and selection coefficients in each introduced population based on census sizes since founding. A single founding population was produced using the following parameters: Ne = 20,000 (based on estimates of GH size from Whiting et al. 2021); μ = 4.89e-8 [(Künstner et al., 2016)](https://paperpile.com/c/U27TGp/5MhYP); r = 1e-8; along a chromosome of 100,000 bp. This population was run until full coalescence was achieved based on tree sequence recording with checkCoalescence=TRUE. This founding population was used to repeatedly seed individuals for each of the four introduced populations with per-generation population sizes set according to observed census sizes accounting for potential error in census estimates. Upon founding, each introduced population was simulated forwards-in-time for 8 (IC and IT) or 7 (IUL, ILL) generations, assuming a generation time of eight months. At each generation, the per-population allele frequencies of all segregating mutations were recorded. Each introduced population was simulated 200 times.

Neutral allele frequency trajectories simulated over the course of the transplant experiment demonstrated an appreciable effect of drift in all introduced populations relative to the founding population over the same time period (Figure S5). As a proxy for the extent of drift, the mean and variance of inferred selection coefficients in IC (mean = 0.127, σ² = 0.012) was greatest followed by IT (mean = 0.114, σ² = 0.010), ILL (mean = 0.111, σ² = 0.009) and IUL (mean = 0.107, σ² = 0.008), in agreement with other demographic analyses. In general, there was reasonable overlap among the distribution of selection coefficients calculated from observed allele frequency change and simulated allele frequency change (Figure S5), demonstrating that much of the observed differentiation between IC, IT, ILL, IUL to their source may be expected under neutrality. Both ILL and IT on average tended to lose or fix standing polymorphisms more readily than IUL and IC (mean proportion of polymorphisms lost/fixed per simulation IC= 16.6%, ILL=16.5%, IUL=15.6%, and IT=14.5%), however the largest values of polymorphisms lost from individual simulations were observed in the tails of IC (max = 27.5%) and IT (max = 26.8%) (Figure S5). These results show that the changes in census size are sufficient to induce the signatures of elevated drift that we observe, in particular in IC and IT.

*Testing for correlation with recombination*

We tested whether any of our outliers were enriched in areas with low or high recombination (Table S21). No detailed recombination landscape exists for guppies, therefore we used a linkage map derived from a F2 cross in Whiting et al. (2021b). We then smoothed the recombination map using the smooth.spline() function in R and manually adjusted positions and orientations to correspond with the genome synteny. We extracted the lower and upper quantiles of recombination rate (0.53 and 9.2 cMb). We tested for significant overlap of these high and low recombination windows with each selection statistic using a permutation test, permuting 1000 times in regionR (Gel et al. 2016).

## **SUPPLEMENTARY FIGURES:**


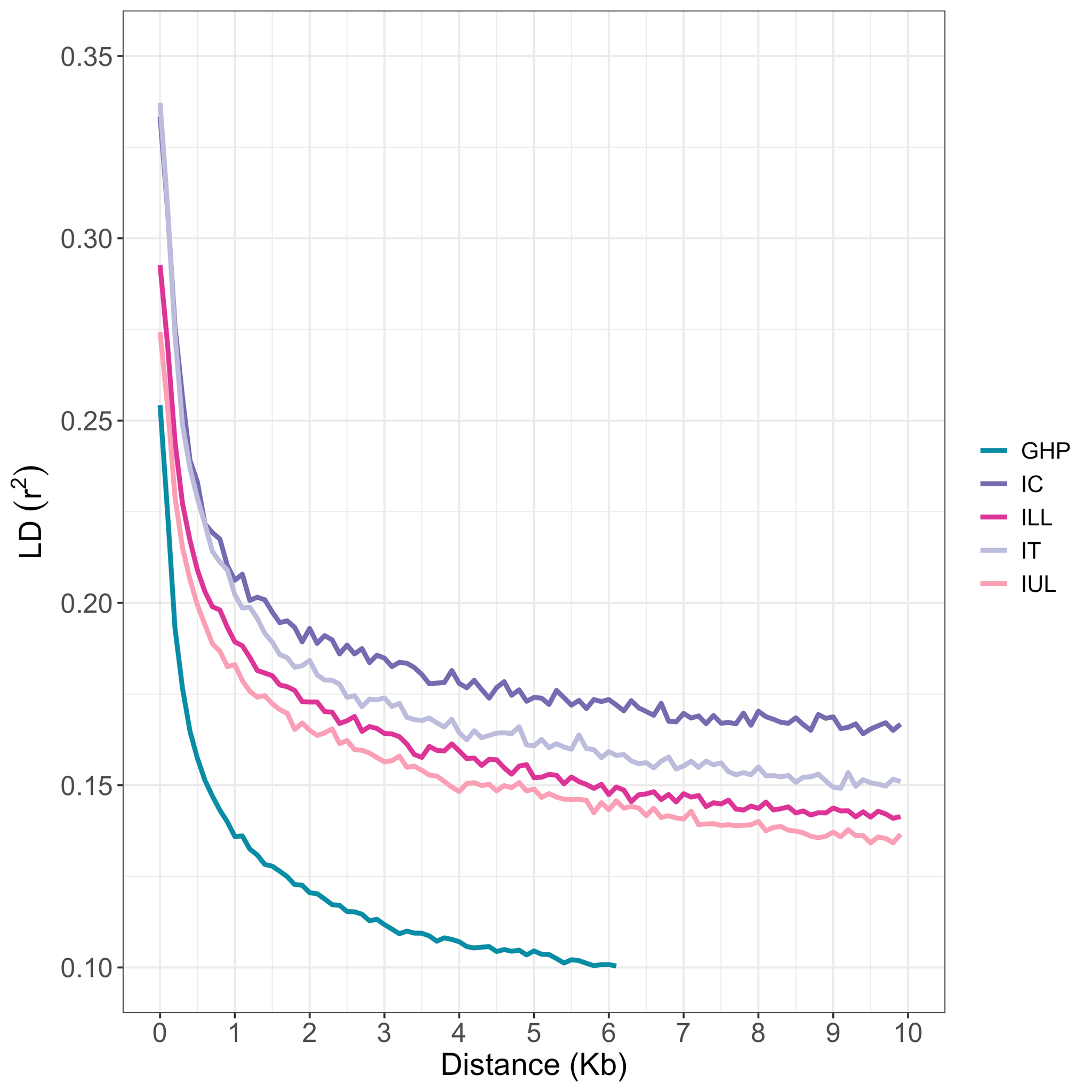


Figure S1: Linkage decay for each experimental population and their source.


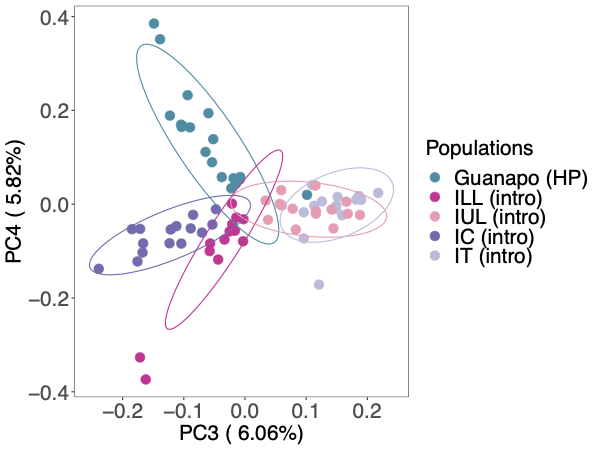


Figure S2: PC3 and PC4 with populations coloured according to river.


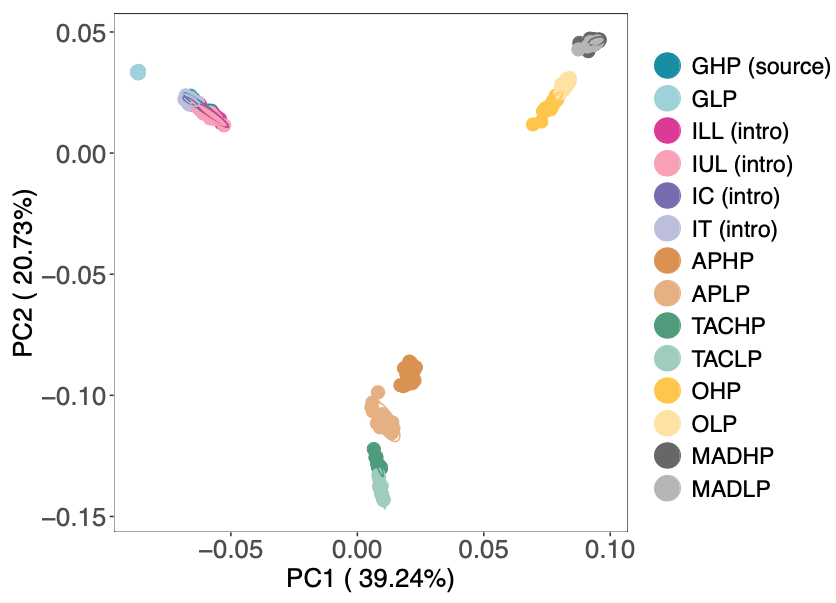


Figure S3. Principal component analysis with natural populations described in [(Whiting et al., 2021a)](https://paperpile.com/c/U27TGp/pSKrq) illustrating the limited population structure between GHP and the introduced populations.


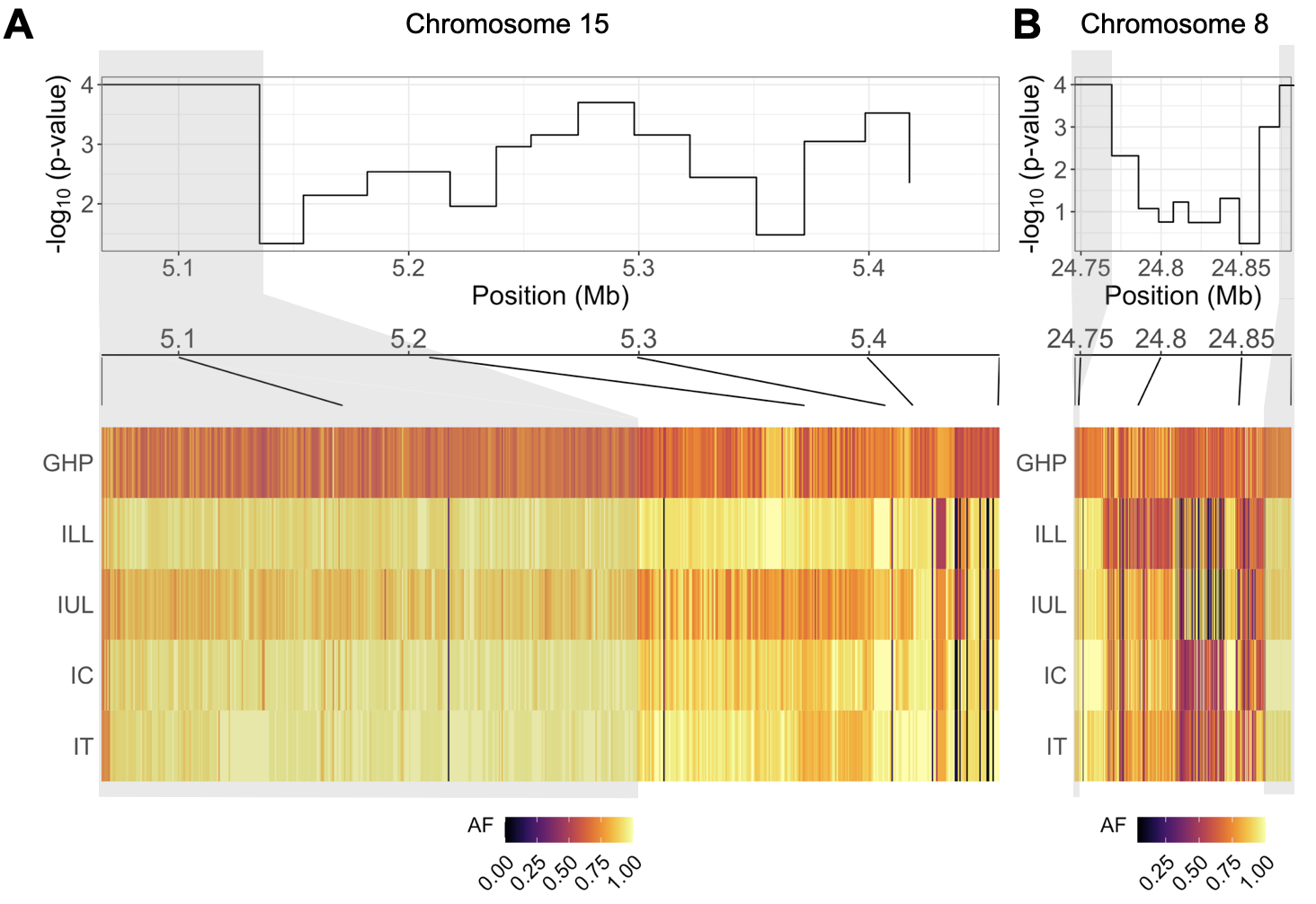


Figure S4: Allele frequency changes compared to eigenvalue p-values for outlier regions. (A) chromosome 15 region, including the region that is an overlapping outlier for haplotype statistics. (B) chromosome 8 region. Both panels show log transformed p-values. In grey are the regions that are outliers in the eigenvector. Bottom panels show allele frequency of each population.


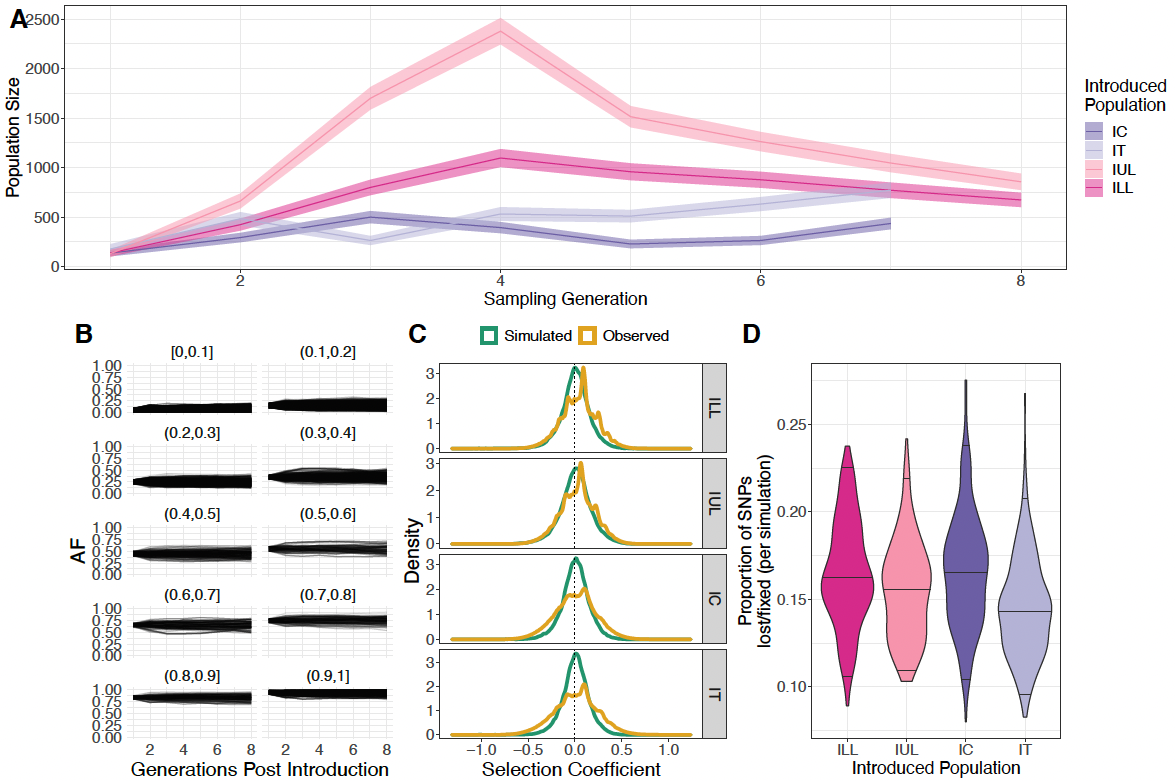


Figure S5: Summary of simulation analysis. Simulated demographies based on census data are shown in (A). Values are means of estimates of the number of males and females with 95% confidence intervals. Examples of simulated allele frequency trajectories (from IC) are visualised in (B) and are grouped according to starting allele frequency. Correspondence between observed and simulated distributions of inferred selection coefficients are shown in (C). Each distribution is composed of selection coefficients calculated from all genome-wide SNPs. Because selection coefficients cannot be calculated for SNPs that are lost or fixed (where final frequency = 0), the probability of loss/fixation is shown in (D). Each violin denotes the median, lower (2.5 %) and upper (97.5%) quantiles for the proportion of SNPs lost or fixed per simulation (total N = 200).


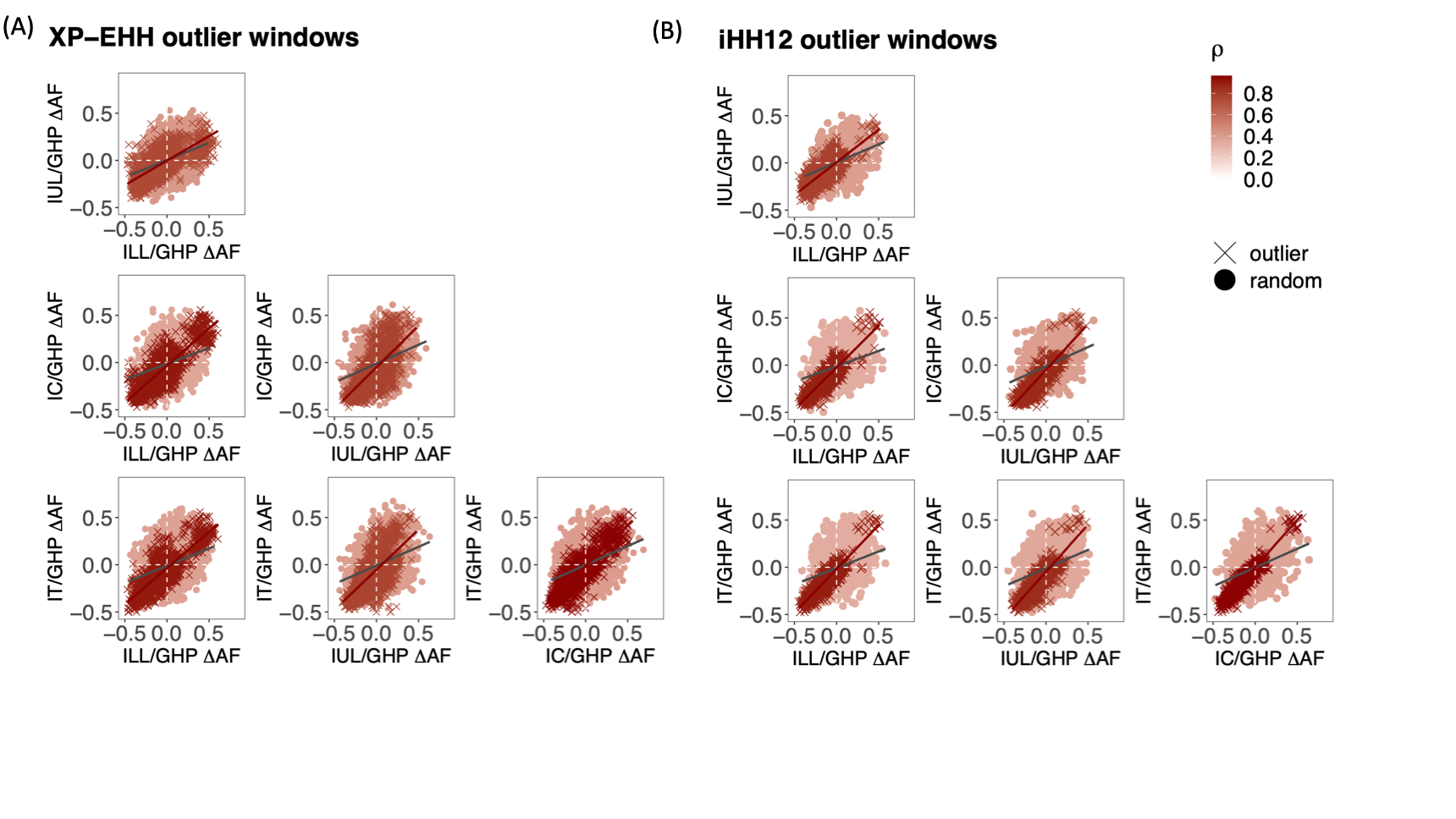


Figure S6: Allele frequency changes in the outlier windows on chromosome 15 for XP-EHH (A) and iHH12 (B). Crosses are allele frequency changes of SNPs in the region’s outlier windows, closed circles are allele frequency changes of a randomly drawn set of SNPs of equal size. Slopes in red are those for outliers while slopes in black are genome-wide.


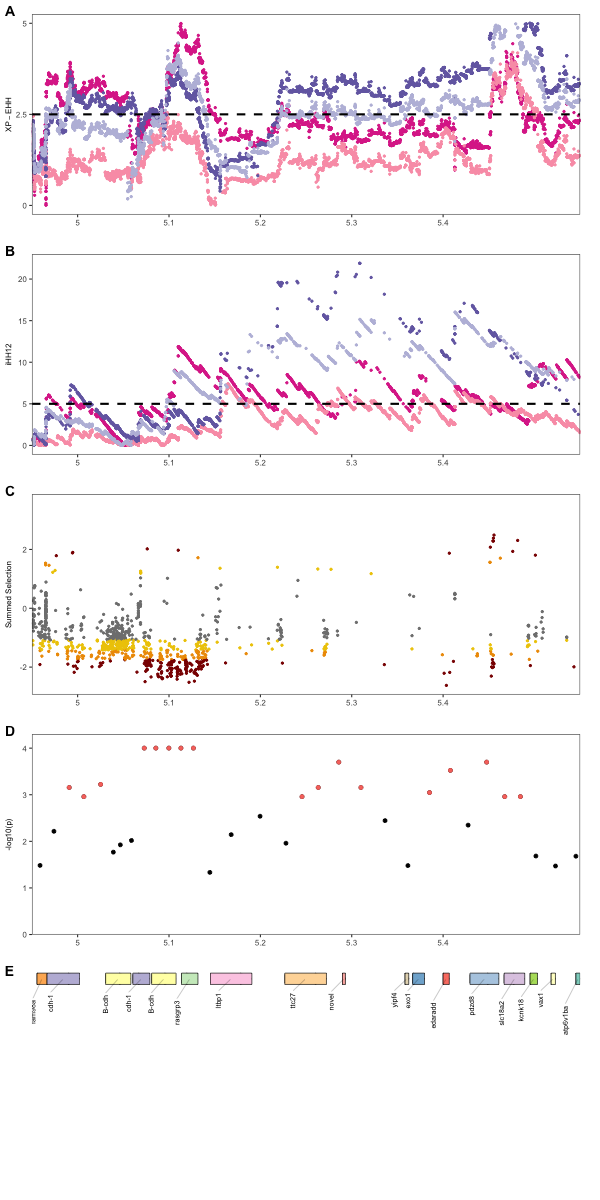


Figure S7: Comparing selection statistics in candidate region on chromosome 15. (A) XP-EHH (B) iHH12 (dashed lines indicate significance cut-off and colours indicate each population, colours as in main text. (C) Summed selection coefficients from each population estimated per SNP. Summed selection coefficients represent the summed estimates of standardised allele frequency change between the source (GHP) and each introduced population. Values are maximised when standardised allele frequency change is large in all populations and allele frequency change is in the same direction (same sign for estimated selection coefficient). Colours indicate SNPs with summed selection coefficients above the 95% (yellow), 99% (orange) and 99.9% quantiles defined by the neutral simulations based on census size estimates (D) transformed p-values per window on the eigenvector analysis (E) predicted gene models.


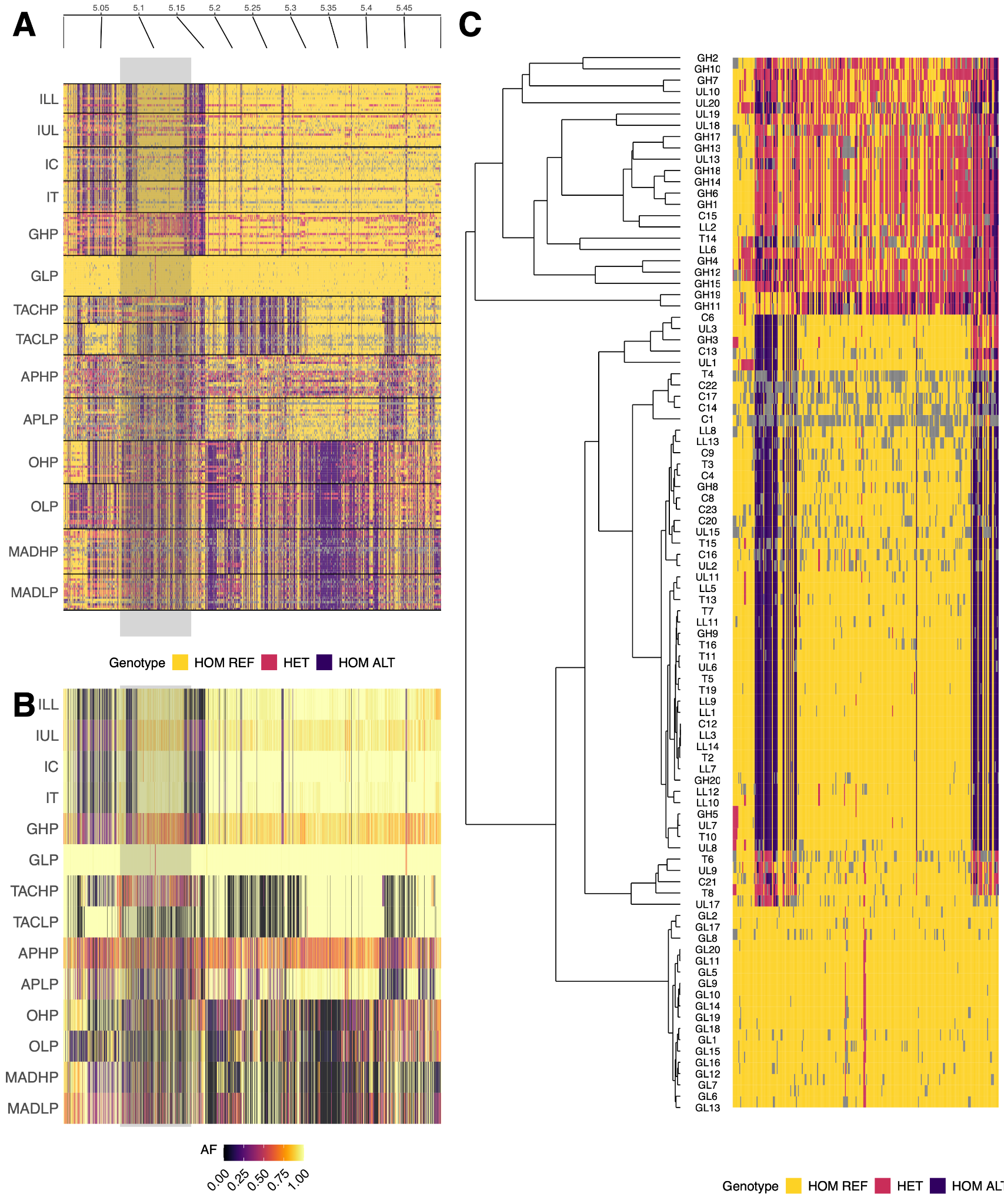


Figure S8: Genotype plots comparing experimental populations to naturally colonised populations in our candidate region on chromosome 15 (5.0 Mb - 5.5Mb) (A) Genotype plots (B) Allele frequency plots. Grey block represents the significant eigenvector windows. (C) clustering plots with genotype plots of experimental populations, source (GHP), and closely related LP population (GLP) of just the grey block. Alleles are polarised to GLP.


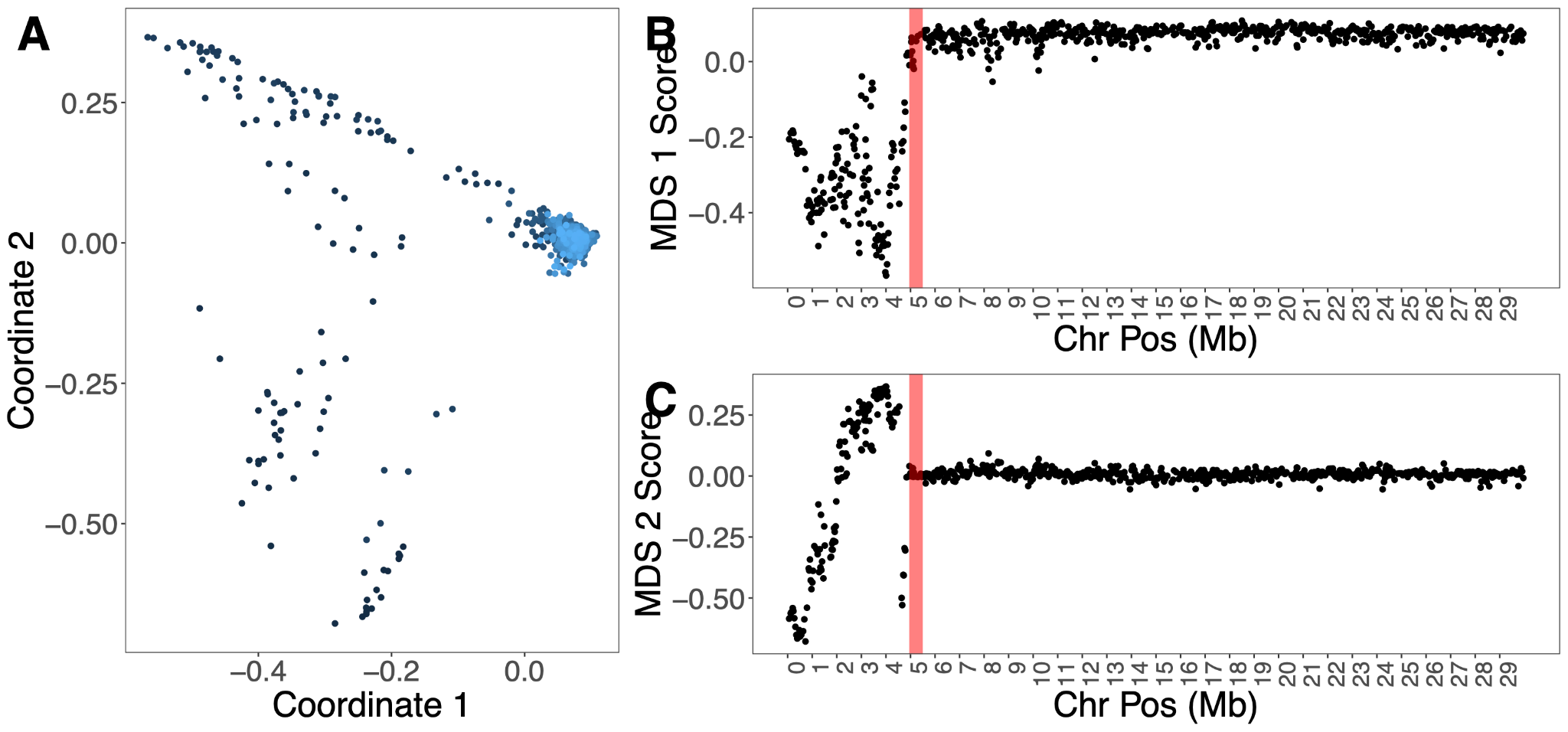


Figure S9: LocalPCA (Li and Ralph 2019) analysis over chr15. Each point in panel A represents a 200 SNP window, with distance between points in MDS space (Co-ordinates 1 and 2) indicative of similar ancestry among windows. The colour of points in panel A represents location along the chromosome, with dark regions at the start and light-blue regions at the end. Extreme values on co-ordinates 1 and 2 are particularly sensitive to inversions, recombination coldspots, and gene density. Panels B and C highlight window scores for MDS1 (B) and MDS2 (C) along chr15, both of which reveal extreme values at the start of chr15. This is consistent with low recombination close to the acrocentric telomere observed on this chromosome and others in the genome (Whiting et al. 2021b). The region of the chromosome highlighted in red represents candidate regions (4.95-5.5 Mb) involved in convergent rapid adaptation among the four introductions, which fall outside of extreme local ancestry region.

**REFERENCES:**

[Andrews, S. (2010). FastQC: a quality control tool for high throughput sequence data.](http://paperpile.com/b/U27TGp/yfWf)

[Browning, S.R., and Browning, B.L. (2007). Rapid and accurate haplotype phasing and missing-data inference for whole-genome association studies by use of localized haplotype clustering. Am. J. Hum. Genet. *81*, 1084–1097.](http://paperpile.com/b/U27TGp/a7rbH)

[Delaneau, O., Marchini, J., and Zagury, J.-F. (2011). A linear complexity phasing method for thousands of genomes. Nat. Methods *9*, 179–181.](http://paperpile.com/b/U27TGp/osnB9)

[Fraser, B.A., Whiting, J.R., Paris, J.R., Weadick, C.J., Parsons, P.J., Charlesworth, D., Bergero, R., Bemm, F., Hoffmann, M., Kottler, V.A., et al. (2020). Improved Reference Genome Uncovers Novel Sex-Linked Regions in the Guppy (Poecilia reticulata). Genome Biol. Evol. *12*, 1789–1805.](http://paperpile.com/b/U27TGp/EDJqQ)

Gel B., Diez-Villanueva A., Serra E., Buschbeck M., Peinado M.A., Malinverni R. (2016). regioneR: an R/Bioconductor package for the association analysis of genomic regions based on permutation tests. *Bioinformatics*, 32:, 289-291.

[Haller, B.C., and Messer, P.W. (2019). SLiM 3: Forward Genetic Simulations Beyond the Wright-Fisher Model. Mol. Biol. Evol. *36*, 632–637.](http://paperpile.com/b/U27TGp/tZkz)

[Howe, K.L., Achuthan, P., Allen, J., Allen, J., Alvarez-Jarreta, J., Amode, M.R., Armean, I.M., Azov, A.G., Bennett, R., Bhai, J., et al. (2021). Ensembl 2021. Nucleic Acids Res. *49*, D884–D891.](http://paperpile.com/b/U27TGp/rrJPX)

[Krueger, F. (2012). A wrapper tool around Cutadapt and FastQC to consistently apply quality and adapter trimming to FastQ files.](http://paperpile.com/b/U27TGp/7rakR)

[Künstner, A., Hoffmann, M., Fraser, B.A., Kottler, V.A., Sharma, E., Weigel, D., and Dreyer, C. (2016). The Genome of the Trinidadian Guppy, Poecilia reticulata, and Variation in the Guanapo Population. PLoS One *11*, e0169087.](http://paperpile.com/b/U27TGp/5MhYP)

[Lencz, T., Lambert, C., DeRosse, P., Burdick, K.E., Morgan, T.V., Kane, J.M., Kucherlapati, R., and Malhotra, A.K. (2007). Runs of homozygosity reveal highly penetrant recessive loci in schizophrenia. Proc. Natl. Acad. Sci. U. S. A. *104*, 19942–19947.](http://paperpile.com/b/U27TGp/nL3ug)

[Li, H., and Durbin, R. (2009). Fast and accurate short read alignment with Burrows-Wheeler transform. Bioinformatics *25*, 1754–1760.](http://paperpile.com/b/U27TGp/o38nq)

LI, H. and Ralph, P. (2019) Local PCA shows how the effect of population structure differs along the genome. Genetics 211, 289-304

[Malinsky, M., Svardal, H., Tyers, A.M., Miska, E.A., Genner, M.J., Turner, G.F., and Durbin, R. (2018). Whole-genome sequences of Malawi cichlids reveal multiple radiations interconnected by gene flow. Nat Ecol Evol *2*, 1940–1955.](http://paperpile.com/b/U27TGp/tKOBw)

[Szpiech, Z.A., and Hernandez, R.D. (2014). selscan: an efficient multithreaded program to perform EHH-based scans for positive selection. Mol. Biol. Evol. *31*, 2824–2827.](http://paperpile.com/b/U27TGp/dM2U)

[Wang, M., Zhao, Y., and Zhang, B. (2015). Efficient Test and Visualization of Multi-Set Intersections. Sci. Rep. *5*, 16923.](http://paperpile.com/b/U27TGp/OqfKX)

[Whiting, J.R., Paris, J.R., van der Zee, M.J., Parsons, P.J., Weigel, D., & Fraser, B.A. (2021a) Drainage-structuring of ancestral variation and a common functional pathway shape limited genomic convergence in natural high- and low-predation guppies.](http://paperpile.com/b/Dv44SY/QNwXf) PLoS genetics, 17(5), e1009566

Whiting, J. R., Paris, J.R., Parsons, P.J., Matthews, S., Reynoso, Y., Hughes, K.A., Reznick, D., and Fraser, B.A. (2021b) On the genetic architecture of rapidly adapting and convergent life history traits in guppies. bioRxiv.
